# Integrating Values and Ethics into Wildlife Policy and Management—Lessons from North America

**DOI:** 10.3390/ani1010126

**Published:** 2011-01-25

**Authors:** Camilla H. Fox, Marc Bekoff

**Affiliations:** 1 Project Coyote, Larkspur, CA 94977, USA; 2 Animal Welfare Institute, Washington, DC 20003, USA; 3 Ecology and Evolutionary Biology, University of Colorado, Boulder, CO 80309-0334, USA; E-Mail: marc.bekoff@gmail

**Keywords:** wolves, reintroduction, ethics, values, wildlife management, predator management, coexistence, attitudes, human-wildlife interaction, carnivores

## Abstract

Few animals provoke as wide a range of emotions as wolves. Some see wolves as icons of a lost wilderness; others see them as intruders. As the battle continues between wolf proponents and opponents, finding solutions that resolve conflicts while supporting the integrity of nature is challenging. In this essay we argue that we need to make room for wolves and other native carnivores who are re-colonizing areas from which they were extirpated. Strategies that foster coexistence are necessary and wildlife agencies must consider all stakeholders and invest adequate resources to inform the public about how to mitigate conflicts between people/domestic animals, and predators. Values and ethics must be woven into wildlife policy and management and we must be willing to ask difficult ethical questions and learn from past mistakes.

## 1. Introduction

Ethics in our Western world has hitherto been largely limited to the relations of man to man. But that is a limited ethics. We need a boundless ethics which will include the animals also.… The time is coming when people will be amazed that the human race existed so long before it recognized that thoughtless injury to life is incompatible with real ethics. Ethics is in its unqualified form extended responsibility to everything that has life.

— Albert Schweitzer, 1924

In the United States, few animals provoke as wide a range of emotions as wolves. For some, wolves are icons of a lost wilderness; their return symbolizes the return of wild nature and the integrity of healthy ecosystems. For others, wolves are viewed as vicious predators with malicious intentions and are better off dead. Such deeply held beliefs about a large carnivorous mammal that was exterminated throughout most of its historic range in the conterminous United States in the nineteenth and twentieth centuries has stirred an impassioned debate that is bound to become even more heated as the U.S. government considers removing wolves from the federal endangered species list and turning management over to the states.

Prior to the arrival of European settlers in the 1600s, wolves existed throughout much of the North American continent. European colonists, however, sought to eradicate wolves and other large carnivores, viewing them as dangerous and bloodthirsty predators and an impediment to progress [1,2,3,4]. As early as the seventeenth century, bounties were placed on wolves by U.S. government agencies, and by the 1930s, gray wolf populations were extirpated from the western United States [2]. A small pocket of wolves remained in the Great Lakes region of Minnesota, despite concerted efforts to eliminate them with poisons, bounties, and intensive trapping efforts [2,5]. Subsequently, public attitudes toward predators gradually changed, and in 1973 wolves received legal protection with the passage of the Endangered Species Act (ESA). With federal protection, wolves began to recolonize northwest Montana, and in 1995 the U.S. Fish and Wildlife Service (USFWS) began a controversial wolf reintroduction program in the Northern Rockies. In recent years, wolf numbers increased in the northern Rocky Mountains and in the western Great Lakes states of Minnesota, Michigan, and Wisconsin.

Deeming the gray wolf adequately recovered, in 2003, the USFWS reclassified gray wolves from endangered to threatened status in the lower forty-eight states (with the exception of the Southwest designated population segment, which remained endangered). The reclassification rule was considered the first step in the eventual elimination of all federal protections for gray wolves in the contiguous states. Animal protection and conservation organizations challenged the ruling, however, arguing that it was premature to remove federal protections for gray wolves and that the USFWS's actions subverted the intent of the ESA to restore listed species to a significant portion of their historic range [6]. In 2005, a U.S. District Court ruled in the plaintiff's favour and overturned the 2003 USFWS rule, restoring the endangered status to gray wolves (except in Minnesota, where they are listed as “threatened” under the ESA). Despite this ruling, the U.S. federal government continues to seek delisting of gray wolves in the lower forty-eight states and animal advocacy and conservation organizations continue to challenge the proposed delisting, arguing that the federal government has failed to develop a comprehensive range-wide strategy for recovering gray wolves [6].

Because the return of the wolf to the conterminous states is so laden with human values, attitudes, and beliefs, we argue that this historical moment presents a unique opportunity for reflection about the ethical issues involved in wolf restoration and the development of practical models for how humans can learn to coexist with wolves in an increasingly humanized landscape. By beginning with an ethical framework and dialogue that considers the interests and values of all stakeholders, including the wolves, who also are entitled to a point of view, we can ensure the process of wolf conservation and management is inclusive and democratic and better serves all affected. We also argue for less invasive and more humane methods of management and control when and where management and control are deemed necessary.

Wolf recovery and conservation requires a sustained commitment toward building human tolerance for the presence of large carnivores. It also requires proactive outreach aimed at educating the public about the vital ecological role wolves [7] and other large carnivores play in maintaining species diversity and the integrity of ecosystems [8,9,10]. Wolves are the consummate keystone carnivore in North America.

If those communities most affected by reintroduction and recovery efforts are to accept wolves and other large carnivores, conservationists must work toward public education and information dissemination to address real and perceived fears held by members of these communities. Integrating ethics into large carnivore recovery also mandates that we listen to community concerns and invest the necessary resources to build tolerance and dispel misinformation. Wolf conservation in general demands a collaborative process among parties who often do not speak to one another. A comprehensive wolf recovery and conservation agenda addresses animal protection, ecological concerns, and socio-political processes.

## 2. Ethical Questions to Ponder

“WOLVES—Government Sponsored TERRORISTS”

—Bumper sticker from www.savethe-usa.com

“A crucial point is that good science rests on good ethics. What scientists do matters; it counts ethically.”

—Jickling and Paquet [11]

“The whale in the sea, like the wolf on land, constituted not only a symbol of wildness but also a fulcrum for projecting attitudes of conquest and utilitarianism and, eventually, more contemporary perceptions of preservation and protection.”

—Kellert [12]

Wolves are a prototypical example of an animal whose reputation precedes them. They bring out extremes in human emotions from almost romanticized idolatry and reverence to blatant contempt and hate (as reflected in the bumper sticker slogan above) that have deep historical roots [1,3,4,13]. Prehistorically, in oral tradition, human fears of wolves and other large carnivores were reflected in fairy and folktales such as *Little Red Riding Hood*, a story in which a wolf follows Little Red Riding Hood home, eats her grandmother, and, according to some interpretations, rapes her. This is a story that is still read to young children throughout the world. Historically, people have viewed wolves as threats to livestock and as competitors in the human hunt for food or sport [1]. As a result of such conflicts, humans are usually the most important cause of mortality of adult wolves and other large carnivores, even within protected areas [14].

Ethical reflection is needed in attempting to recover wolf populations on lands where abundant domesticated prey (*i.e.*, unprotected livestock on the open range) bring them into conflict with livestock [4,5]. Can we really blame them for taking advantage of an accessible meal? Should we be moving predators around if we cannot let them be the animals that they have evolved to be, when recovery means intensive management, or when the areas into which we place them are increasingly developed, fragmented, and hostile? Can we call wolf recovery a success in the United States when we have confined recovery efforts to less than 5 per cent of the wolf's historical range and when approximately 80 per cent of all known wolf mortalities in the tri-state area of Montana, Idaho, and Wyoming are intentional removals by the U.S. federal government?

## 3. Some Guiding Principles: The Importance of Ethics

The authors' guiding principles for how we interact with other animals are simple and straightforward: do no intentional harm, treat all individuals with respect and compassion, and recognize that all animals have intrinsic value or worth, irrespective of their utility to other animals, including humans. We recognize and acknowledge that our ethical principles and framework reflect not only our cultural backgrounds, biases, and education but also our deeper Greek-Roman ethical heritage dating back to Socrates, Plato, and even earlier to Indo-European cultures. Ethical positions within human societies differ profoundly across cultures and time. Hence, when we speak of our guiding ethical principles, we do so knowing that they reflect only a few cultural perspectives amongst a broad array of perspectives that come into play when discussing wolf recovery and conservation.

While very few people in any culture attempt to cause intentional harm or delight in doing so in their efforts to conserve and restore ecosystems and biodiversity, the other principles that call for treating individuals with respect and compassion and recognizing an individual's intrinsic value or worth are all too easily overridden because they are too difficult to consistently adhere to regardless of cultural biases. In some cases, while it clearly is not one's intention to cause harm to other animals, the very design of some studies or perhaps the very reality of some conservation efforts means that inevitably some animals will suffer or die. We must ensure that we do everything we can to minimize pain and suffering and cause the least amount of harm.

The recognition that wolves and other individual animals have intrinsic value demands that we consider ethics when we conduct projects and practices that impact them. When we use the term “ethics,” we are referring to Socrates' notion of ‘how we ought to live’ [15]. Hadidian *et al.* [16] also note: “ethics is a conversation about the moral values that inform (or should inform) our thoughts and actions … ethics is not only a critique of who we are as individuals and a society today, it is a vision of what our future may be if we act with ethical sensibilities in mind … ethics is meant to help us refine our knowledge and action, to distinguish better from worse arguments, methods, data and facts.” While many agree that ethics must play a central role in any project involving the use of animals [11,16,17,18], it is interesting to note that in many books on human–animal interactions and carnivore conservation there is often no mention of ethics. This needs to change.

We assert that recovery and conservation efforts for wolves and other carnivores should be firmly rooted in ethical principles. And yet, when we look at current wolf management in the United States, consideration of ethics is largely ignored. For example, the United States Forest Service is planning to ease restrictions on killing predators in protected wilderness areas within the western United States, allowing expanded use of aerial gunning and certain poisons [19]. And the USFWS issued lethal control permits to the states of Wisconsin and Michigan that authorize officials to kill up to fifty-four gray wolves annually if the wolves are perceived as threatening livestock or pets [6]. However, animal and environmental organizations sued to stop the killing and in August 2006 the federal court ruled in the plaintiffs' favour, stating that the issuance of lethal kill permits violates the ESA. The state of Wisconsin argued the kill permits were “necessary to maintain social tolerance for the wolves” [20]. In her court decision, the judge responded by saying, “The recovery of the gray wolf is not supported by killing 43 gray wolves” [20].

Furthermore, there are examples of “Judas wolves” [21], individuals who are collared and then followed back to their pack so that other pack members can be located. The Judas wolf, having unknowingly betrayed its pack-mates, is then killed along with the entire pack, including pups. Despite the fact that gray wolves remain federally listed under the ESA, more than three hundred have been killed by the U.S. federal government since 1987, most for preying on livestock [21]. Lethal removal of wolves continues while we know, and have known, that eliminating predators does little to increase economic gains for livestock ranchers [22] or to reduce attacks over the long-term [23].

Discussions about ethics and animals can make people uncomfortable. Ignoring questions about our ethical responsibilities to animals not only compromises their lives and our integrity but also can compromise the quality of scientific research. More and more students and practicing scientists recognize that asking questions about ethics is in the best interests of “good science,” and increasing numbers of non-researchers are also keenly interested in animal well-being [24,25,26,26,27,28,29,30,31,32]. Wildlife managers and scientists are under growing scrutiny by a concerned public who not only question how funds are used to support wildlife management practices and various scientific research projects but also want wildlife managers and scientists to be less arrogant and authoritarian and more accountable to those who support them [12,26,29,32,33,34]. Furthermore, science, including conservation biology, is not value-free [11,18,32,35]. Soulé [35] argued that conservation biology must be based on a set of ethical axioms. Personal views held by scientists influence funding and the dissemination (or withholding) of certain results. Indeed, dealing with personal sentiments and emotional conflicts makes questions about what we ought to do extremely difficult. Complicating the situation is the fact that values and sentiments change with time and are sensitive to demographic, political, and social-economic variation, as well as to personal whims. However, regardless of changes in values and sentiments, if we remain loyal to doing no intentional harm, treating all individuals with respect and compassion, and recognizing that all animals have intrinsic value and worth irrespective of their utility (the authors' guiding principles expressed above), we will ensure high ethical standards in our discussions on interaction with other species and in our actions which impact them.

## 4. Considering all Perspectives

As we try to repatriate and restore wolves to the landscape, we have a duty to consider the broad impacts of such efforts from all angles: on the wolf packs, the populations and ecosystems from which they are taken, and on the human, animal, and ecological communities in which they are placed. In discussing the social dynamics affecting wolf conservation in Yellowstone National Park, for example, Clark *et al.* [36] aptly state, “Understanding the human participants is essential to understanding what has happened, why, and what is likely to happen.” While it is imperative to consider and negotiate differing perspectives and values amongst various human stakeholder groups in wolf recovery efforts, we contend one viewpoint is often missing in this discussion: the wolf's. We focus on under-represented perspectives in wolf-recovery efforts (e.g., the wolf's viewpoint) and does not attempt at understanding the viewpoints of all interest groups. The growing body of literature on animal cognition and emotions demonstrates undeniably that animals have interests and points of view [29,30,34,37]. Like us, they avoid pain and suffering and seek pleasure. They form close social relationships, cooperate with other individuals, and likely miss their friends when they are apart [29,34,37,38]. Emotions have evolved, serving as “social glue,” and playing major roles in the formation and maintenance of social relationships among individuals [37]. Emotions also serve as “social catalysts,” regulating behaviours that guide the course of social encounters when individuals follow different courses of action, depending on their situations [29,30,34,37,39]. If we carefully study animal behaviour, we can better understand what animals are experiencing and feeling and how this factors into how we treat them.

Recognizing that wolves and other animals have emotional lives forces us to consider their needs and interests as individuals, as families, and as members of a community. Because the wolf is a species with complex social structures and tight family bonds, we must consider the ethical implications of our actions when we disrupt family packs through management and control programs. We need to consider the wolf's point of view in our overall conservation and recovery efforts.

## 5. Wolf Persecution: Repeating the Cycle?

Consider the case of the Mexican wolf reintroduction program. Mexican wolves once ranged from central Mexico up into Arizona and New Mexico [40]. They were exterminated throughout most of their historic range by the U.S. Bureau of Biological Survey and its successor agency, the U.S. Department of Agriculture Animal Damage Control program (now called “Wildlife Services”) [41]. In 1976, the subspecies was placed on the endangered species list and a reintroduction effort was initiated in 1998. While approximately 90 captive wolves were reintroduced over the course of eight years in New Mexico and Arizona, as few as 35 (estimated range: 35–49; mean estimate: 42; USFWS 2006c) wolves remained in the wild population by the end of 2005 [42]. From 1998 through 2005, illegal shooting (23), lethal agency control (3), vehicle collisions (9), and capture complications (1) accounted for the human-caused deaths of 36 wolves; and 83 wolves were captured and either removed or translocated at the agencies' discretion for management purposes, which included 31 wolves involved in livestock losses (Adaptive Management Oversight Committee AMOC [42,43,44]). High wolf “failure rates” (mortalities + removals) are precluding population growth, causing population declines in 2004 and 2005, despite continued releases of wolves during those years [43,44]. The program has been criticized for poor management, bureaucratic processes that hinder effective recovery, and unrealistic political boundaries that do not allow wolves to colonize public lands outside of the defined recovery zones [5,40]. Moreover, ranchers are not required to improve or alter their livestock husbandry practices to reduce predation even after a wolf is removed or killed (which is the case throughout the United States, not just in the Mexican wolf reintroduction program). And, in July 2006, the USFWS announced its acceptance of a set of recommendations that, if implemented, will allow the government, tribes, and private individuals to trap or kill Mexican wolves with few restraints when the combined populations in New Mexico and Arizona exceed 125 wolves [44], a cap that cannot be considered either viable over the long term or ecologically effective for the region [10,28]. We simply must ask, “What are we doing and why are we doing it?” This sort of bureaucratic mismanagement and shameless killing must be stopped if we are ever to extricate ourselves from the persecute/eliminate/try-to-recover-the-species cycle. How can we get out of this loop and constructively facilitate coexistence?

## 6. Trade Offs: Individuals *vs.* Species

In conservation biology, the interests and rights of individuals are sometimes traded off against perceived benefits that accrue to higher levels of organization: populations, species, and ecosystems. Animal protection advocates who prioritize the welfare of individual animals are often marginalized because their perspectives are perceived as obstacles to conservation efforts. Estes [45] poignantly and succinctly gets to the heart of the matter in his discussion of whether or not to rehabilitate oiled wildlife, specifically California sea otters (*Enhydra lutris*):

The differing views between those who value the welfare of individuals and those who value the welfare of populations should be a real concern to conservation biology because they are taking people with an ostensibly common goal in different directions. Can these views be reconciled for the common good of nature? I'm not sure, although I believe the populationists have it wrong in trying to convince the individualists to see the errors of their ways. The challenge is not so much for individualists to build a program that is compatible with conservation—to date they haven't had to—but for conservationists to somehow build a program that embraces the goals and values of individualists because the majority of our society has such a deep emotional attachment to the welfare of individual animals.… As much as many populationists may be offended by this argument, it is surely an issue that must be dealt with if we are to build an effective conservation program.

Some of the main issues concerning trade-offs among individuals, populations, species, and ecosystems are highlighted when considering reintroduction programs. Such efforts raise questions about when and whether it is permissible to override an individual's life for the good of its species—when can individuals be traded off for conservation gains? Consider the reintroduction of gray wolves into Yellowstone National Park (YNP). All of the wolves who were reintroduced into YNP were translocated from Canada. Some were separated from their family packs; some died shortly after their release [4]. Currently, those that venture out of the protective zones of YNP may be lethally removed if they prey on livestock. Our view is that individuals count and that jumping among different levels of organization is not as seamless as some make it out to be. We believe that carnivore recovery programs are essential to restoring ecosystem integrity and diversity, but we also believe that in so doing we must be rigorous in the questions we ask, mindful of the individual animals we are translocating and of their progeny, and ethical in the way we conduct such programs. Researchers have an obligation to attempt to fully understand the effects of reintroduction programs on life history strategies, demography, behaviour, and animals' lives [18].

## 7. Reintroduction *vs.* Natural Recovery: The Role of Fear

Recovering native species through reintroduction programs requires massive human effort and large sums of money. Humans and human society are major factors in what goes right or wrong, and people who are most affected at the local level are sometimes resentful and hostile at having to share land and space with a large predator that their forefathers purposefully eradicated. This is easy to understand especially when they have been living their lives and making their livelihoods in the absence of these predators. Moreover, the myth of the savage wolf persists and this also makes it difficult for some people to accept their presence. Fear is a powerful motivator, so those who advocate the reintroduction of wolves must work toward alleviating unfounded concern about their danger, allocate the necessary resources to build tolerance for wolves through public education and outreach programs, and help reduce conflicts where real conflicts exist.

Natural recovery of wolves also presents challenges as seen in Minnesota, Michigan, and Wisconsin; but perhaps more people would be open to the presence of wolves if they return on their own. Those less receptive would be given more time to get accustomed to the fact that wolves are on the way, and those who dislike government intervention might be open to wolves if there were less bureaucratic interference. Yet there's no denying that wolves—whether from reintroduced or naturally recolonizing populations—face tough odds when attempting to venture beyond the political boundaries in which they've been confined. For example, in September 2006, a wolf likely dispersing from one of the Yellowstone or central Idaho packs was found dead in a leghold trap on private land in Utah [46]. Four years earlier, another wolf was discovered in the state—also found in a leghold trap [46]. In Maine and Vermont where gray wolves historically roamed, at least three wolf-like canids believed to have dispersed from Canada have been shot or trapped before their presence in the states was even acknowledged [47]. So a high tolerance level among the general public does not necessarily translate to safety for wolves if a few key humans (e.g., trappers, hunters, ranchers) have low tolerance; thus, dispersing wolves often find a lethal human environment where basic survival becomes a challenge. While there is certainly no guarantee that natural recovery will increase tolerance for wolves over reintroduction programs, the costs and benefits of both should be weighed before recovery efforts are implemented.

We also need to reconcile the disparity in the status of wolves who are reintroduced and those who appear on their own. The former are granted “experimental, non-essential status” under section 10(j) of the ESA and are subject to being killed for being the predators that they are (when they predate livestock), whereas naturally occurring individuals are ostensibly granted full protection under the ESA. While some argue reducing federal protections for reintroduced wolves was a necessary concession to garner acceptance from the ranching community [4], we must ask if it is acceptable to continue to designate wolves “experimental, non-essential” and then kill them when they prey on livestock while not requiring ranchers to take some responsibility to reduce losses by removing livestock carcasses and improving their animal husbandry techniques. Caring properly for livestock is and should be one of the costs of doing business and should be reflected in the price of meat at the supermarket. Unfortunately, the current system in the United States externalizes the costs of livestock predation, and it is the American taxpaying public that bears these costs through subsidies for government predator control programs and livestock grazing subsidies. The wolves also pay with their lives when they are lethally “removed” for preying on livestock.

## 8. The Future of Wolf Conservation and Management in the United States

In the United States, as the federal government evaluates the opportunity to delist wolves, we can expect the debate about wolf conservation and management to intensify with ethics and human–wolf conflict mitigation moving front and centre to the debate. When delisting occurs, wolves will no longer be federally protected under the Endangered Species Act; management will revert to the states and tribes. Heated debates have already begun about how wolves will be managed and whether traditional forms of management, including trophy hunting and commercial and recreational fur trapping, will be allowed, as they are for some species of large carnivores. For example, Minnesota's state management plan would allow wolves to be killed to protect domestic animals, even if attacks or threatening behaviour have not occurred, and eventually allow for the commissioner to “prescribe open seasons” on wolves, thereby legalizing trophy hunting and fur trapping [48]. The Minnesota state law also allows for paying “certified gray wolf predator controllers” $150 for each individual killed (see Section 97B.671 Predator Control Program of Minnesota State Law for details).

Wyoming's proposed management plan calls for wolves to be classified with “dual status” (Wyoming Game and Fish Department (WGFD) 2003; more details can be found at http://www.sublette.com/examiner/v2n34/draftwolfplan.pdf), allowing them to be managed as trophy game in national parks and wilderness areas and as a “predatory animal” outside of these designated areas, allowing them to be killed at any time. The USFWS, however, has rejected Wyoming's plan, stating it is inadequate to ensure long-term viability of wolf populations [49]. Despite this, in January 2007, the Wyoming legislature introduced a bill that would authorize the killing of almost two-thirds of the wolves in the state. Wildlife advocates are challenging both the delisting process and the state management plans, which has served to increase public debate about the future of wolf management in the United States [6].

One need only look at Alaska to see why there is significant concern about how wolf management may unfold in the lower forty-eight states. In Alaska, wolves are not considered endangered and receive none of the legal protections under the ESA that their counterparts do in the rest of the United States. They can be legally trapped, trophy hunted, and aerially gunned where they are chased to exhaustion by low-flying aircraft and then shot. Between 2003 and 2006, more than 550 wolves have been killed through aerial gunning in Alaska, despite the fact that Alaskans have twice voted to ban the practice (1996 and 2000) in state-wide ballot measures (the Alaska legislature then overturned those bans). In some areas, the Alaska Board of Game has approved the killing of up to 75 per cent of the wolf population, ostensibly to boost moose and caribou populations for big-game hunters. In 1998, a citizens group called “Alaskans Against Snaring Wolves,” sought to prohibit the use of snares for capturing wolves through an unsuccessful public ballot initiative after photos of severely injured snared wolves were published in local and national media outlets. The grassroots effort and the ensuing public debate it generated on the use of snares and other control methods supported by the Alaska Board of Game highlighted the growing controversy over the ethics of wolf management and individual management techniques, and the way that management decisions are made.

Some have argued that decisions made in Alaska regarding wolves cannot be compared to decisions made in the lower forty-eight states. However, when states like Idaho take an official position that the federal government must forcibly remove all wolves from the state (adopted as House Joint Memorial No. 5 in 2001) and Wyoming wants to declare open season on wolves, it becomes apparent that a similar, firmly rooted anti-wolf sentiment amongst some sectors of the public is not limited to Alaska.

## 9. Integrating Ethics into Wolf and Carnivore Conservation

While strong anti-wolf sentiments persist in some areas of the United States, particularly in more rural regions, such attitudes are rapidly changing as the populace becomes more urban and educated [12,50]. Over the last century, we have seen a shift in the public's attitudes toward wildlife and nature, moving from a primarily dominionistic/utilitarian valuation toward one that is more humanistic/moralistic oriented [12,50,51]. With this shift in public values has come an increased demand for humane, socially acceptable, and ecologically sound management strategies for addressing conflicts between people and wild animals [52,53,54,55]. One national study on public attitudes toward wildlife management concluded that a majority of Americans favour the use of non-lethal methods over lethal in managing wildlife [55]. In this study, survey respondents were asked to rank the importance of factors to be considered when selecting management techniques; human safety, animal suffering, effectiveness, and environmental impacts ranked highest. Less important was monetary cost, suggesting a willingness amongst the public to invest more money to develop methods that ensure public safety and mitigate animal suffering. If lethal controls must be employed, the public would like those methods to be humane and selective [52,55]. Yet one study that looked at lethal carnivore management programs across the globe found that between 30 and 81.3 per cent of the carnivores killed in control operations bore no evidence of involvement in conflicts [56], despite the efforts to target so-called ‘problem animals.’

Strong objections to U.S. government-funded lethal predator control programs have also been expressed by professional scientists with the American Society of Mammalogists (ASM). In 1999, the ASM passed a resolution stating that the “common methods of predator control are often indiscriminate, pre-emptive, lethal measures, particularly in relation to state- and federally funded livestock protection programs … and often result in the needless killing of animals that are not contributing to the problem, as well as many non-target species” [57]. They called on the U.S. Department of Agriculture's Wildlife Services Program and other federal and state wildlife management agencies to “cease indiscriminate, pre-emptive, lethal control programs … and to focus on the implementation of non-lethal control strategies, compensatory measures, and sound animal husbandry techniques” [57].

If ethics, societal values, and animal welfare are not fully vetted and incorporated into wildlife management policies and programs, what are some potential consequences? Increasing use of the public ballot initiative process is one possible outcome if a large segment of the public continues to feel their values and opinions are not considered in decision-making processes. Similarly, if wolf opponents feel their concerns and values continue to go unheard, we may see an increase in illegal killings as have been documented in Idaho where a number of wolves were intentionally poisoned with the deadly poison Compound 1080 after wolves were reintroduced in the region [43].

A first step toward mitigating reactionary responses to wolf conservation policies and practices is for state and federal wildlife agencies to create greater opportunities for public participation in the decision-making process. In the United States, many state and federal wildlife management agencies have been criticized as operating in bureaucratic, self-serving ways that ensure their continued control and power over wildlife management while largely excluding the public from meaningful participation [36]. These institutions often fail to change strategies and policies to reflect new and more holistic ecosystem approaches to wildlife conservation that incorporate adaptive management practices [36,40]. They also tend to shun discussion or consideration of ethics, public attitudes, and values by deeming such concerns as unscientific and contrary to traditional approaches to wildlife management. The current problems with the Mexican wolf reintroduction program reflect this bureaucratic institutional system that largely disregards public input, particularly from the conservation and animal protection communities, and fails to ensure transparency in its processes, policies, and practices [5,40].

So, what is the solution to this entrenched systemic problem? As Clark *et al.* [36] state, “Expanding confused bureaucracies is not the answer, although this is what we often do.… To improve wildlife conservation, especially large carnivore management, bureaucracies must be reformed.” A first step toward wildlife management agency reform is to create models and processes that promote integration and inclusion—where people feel heard, where they feel their values are considered, and where they feel they can have a meaningful say in the matter. Such civic-minded processes will also help foster mutual understanding and common ground and counter the dominant wildlife management paradigm in the United States that tends to promote divisiveness instead of cooperative problem-solving [36].

## 10. Practical Models of Carnivore Coexistence

In addition to new modes of civic processes that foster inclusion and integration, we also need practical on-the-ground carnivore coexistence model programs that promote large carnivore conservation and cooperative community-based problem solving. Clark *et al.* [36] call this “practice-based improvements,” the application of which use actual experience and adaptive management practices to address site-specific conflict areas rather than theoretical principles as the basis for making improvements. Musiani and Paquet [58] argue that such efforts should focus on rural areas where human–wolf conflicts are more likely to occur. We argue that such programs should also incorporate ethics and humane concerns. Globally an increasing number of “practice-based improvement” models provide examples of practices that foster large carnivore conservation and promote coexistence. For example, in Bulgaria, non-governmental organizations have implemented a program aimed at reducing conflicts between livestock and wolves non-lethally and building tolerance for the presence of wolves by supplying shepherds with Karakachan guarding dogs [59]. They have also conducted a broad public awareness campaign that includes outreach to ranchers, students, and the general public [60]. In Sweden, a government-run program provides ranchers with financial support to implement electric fencing and other non-lethal predation deterrents [61]; ranchers are compensated for the presence of carnivores on their property at pre-determined rates, fostering better animal husbandry and carnivore conservation [62]. To date, the program appears to have been successful in reducing losses and building tolerance for the presence of wolves and other large carnivores [61,62]. In Ethiopia, the Ethiopian Wolf Conservation Program employs people from the local communities to protect the wolf, conducts outreach to ranchers to improve livestock and agricultural practices, vaccinates domestic dogs to help prevent the spread of canid diseases, and has an extensive educational program aimed at building local understanding of the important role that the wolf plays in the Bale mountain ecosystem [63].

Isolated models of carnivore coexistence programs that integrate ethics and ecological concerns are beginning to appear in the United States as well. For example, in Marin County, California, a non-lethal cost-share program funded by the county provides qualified ranchers with financial assistance to implement non-lethal deterrents including guard dogs, llamas, improved fencing, and lambing sheds [64,65]. Importantly, the program was adopted as a result of public opposition to the use of poisons, snares, and other lethal methods employed by a taxpayer-subsidized government trapper under the USDA-Wildlife Services program [66]. The debate centred around ethics, animal welfare, and the use of taxpayer monies to support the killing of native carnivores to protect ranching interests. The program has garnered national attention, and initial data from the County Agricultural Commissioner's office indicate it has been effective at helping to reduce livestock losses for a majority of participating ranchers [67,68,69].

Hence, new models of predator/livestock coexistence strategies combined with traditional techniques that historically proved effective in many parts of the world, such as shepherding and the use of guard dogs, have the potential to improve wolf conservation efforts globally [58,62,70].

## 11. Looking to the Future and Learning from the Past: We Can Always Do Better

As conservationists struggle to stem the hastening global biodiversity crises, we face many ethical challenges. How do we balance the urgent need to restore ecosystem health through large carnivore recovery with our obligation to consider ethics and animal well-being? These are difficult questions with no simple answers. Nonetheless, serious ethical reflection, public education, and dialogue are needed before deciding to restore a previously extirpated species such as the wolf. Ultimately, it is unlikely that a quick fix is the best way to proceed, especially when a lack of understanding of the complex and interrelated socio-political, economic, and ecological variables involved can make or break a recovery project. For example, the very early stages of Canada lynx reintroduction into southwestern Colorado were marred by the death of four reintroduced individuals soon after they were released because there was not enough food [18]. Some state officials, independent wildlife biologists, and animal advocates had argued that the available data suggested that the habitat was unsuitable to support viable lynx populations; yet lynx were released using what some called a “dump and pray” strategy [18]. The hasty and politically motivated “quick fix” clearly did not work; however, when reintroduction protocols were changed and attention was given to the scientific data concerning food availability and habitat suitability, fewer deaths by starvation resulted, and ultimately some of the reintroduced lynx went on to breed.

As we attempt to restore wolves and other large carnivores in a human-dominated world where fragmentation—environmental and spiritual—and accelerating urban sprawl threaten to undermine such efforts, it would behove us to look back on history and gauge where we have come from and where we are going. Less than sixty years ago, the last remaining Mexican wolves in Mexico were eliminated by the very same agency that is leading the wolf recovery effort in the United States today; less than thirty-five years ago, wolves were hunted without restrictions in many states [2]. What have we learned since then?

Aldo Leopold who is considered by many as the father of wildlife conservation in North America had an epiphany watching a wolf die (after having slaughtered this one and many others himself), and for the first time connected with an individual wolf in a way he had never experienced before. Through this experience, Leopold stepped beyond seeing the world from a myopic anthropocentric lens and recognized that another species had its own wants and needs—its own intrinsic worth—and a desire to live free and unfettered. Out of this and other experiences, Leopold [71] developed what he termed “The Land Ethic.” In his words:

The land ethic simply enlarges the boundaries of the community to include soils, waters, plants, and animals, or collectively: the land—and it affirms the right of all to continued existence. The extension of ethics to land and to the animals and plants which is an evolutionary possibility and an ecological necessity. In short, a land ethic changes the role of Homo sapiens from conqueror of the land-community to plain member and citizen of it. It implies respect for his fellow-members, and also respect for the community as such.

Ultimately Leopold's Land Ethic was a call to action to create a new paradigm for the way we interact with and coexist with native carnivores—indeed all living beings—one that recognizes the ecological importance of these other species and life forms as well as their intrinsic value. As we struggle to rectify the wrongs of our past and as we gauge our almost limitless power to both create and destroy—and then recreate, restore, and recover other species and ecosystems, we must, like Leopold, take a long moment to reflect upon our actions. We must be willing to ask difficult ethical questions and learn from our past mistakes. Ultimately, we must always challenge ourselves: should we be doing what we are doing and, if so, can we do it better?

Michael Soulé, a founder of the field of conservation biology, perhaps said it best:

We're certainly a dominant species, but that's not the same as a keystone species. A keystone species is one that, when you remove it, the diversity collapses; we're a species that when you add us, the diversity collapses. We can change everything, dictate everything and destroy everything [72].

Soulé is right. As big-brained and often self-centred and arrogant mammals, we can do anything we want anywhere, anytime, and to any other beings or landscapes. We must recognize that this unprecedented power comes with enormous and compelling ethical responsibilities to do the best we can. Let us remember that in most cases we can do better; and in all cases we have an obligation to strive to do better than our predecessors.
